# ^1^H NMR-Based Metabolomics Study of the Toxicological Effects in Rats Induced by “Renqing Mangjue” Pill, a Traditional Tibetan Medicine

**DOI:** 10.3389/fphar.2017.00602

**Published:** 2017-09-04

**Authors:** Can Xu, Caidan Rezeng, Jian Li, Lan Zhang, Yujing Yan, Jian Gao, Yingfeng Wang, Zhongfeng Li, Jianxin Chen

**Affiliations:** ^1^Department of Chemistry, Capital Normal University Beijing, China; ^2^Research Center of Chinese and Tibetan Medicine, Medicine College of Qinghai University Xining, China; ^3^School of Preclinical Medicine, Beijing University of Chinese Medicine Beijing, China

**Keywords:** “RenqingMangjue” pill, ^1^H NMR, metabolomics, toxicity, ICP-MS

## Abstract

“RenqingMangjue” pill (RMP), as an effective prescription of Traditional Tibetan Medicine (TTM), has been widely used in treating digestive diseases and ulcerative colitis for over a thousand years. In certain classical Tibetan Medicine, heavy metal may add as an active ingredient, but it may cause contamination unintentionally in some cases. Therefore, the toxicity and adverse effects of TTM became to draw public attention. In this study, 48 male Wistar rats were orally administrated with different dosages of RMP once a day for 15 consecutive days, then half of the rats were euthanized on the 15th day and the remaining were euthanized on the 30th day. Plasma, kidney and liver samples were acquired to ^1^H NMR metabolomics analysis. Histopathology and ICP-MS were applied to support the metabolomics findings. The metabolic signature of plasma from RMP-administrated rats exhibited increasing levels of glucose, betaine, and creatine, together with decreasing levels of lipids, 3-hydroxybutate, pyruvate, citrate, valine, leucine, isoleucine, glutamate, and glutamine. The metabolomics analysis results of liver showed that after RMP administration, the concentrations of valine, leucine, proline, tyrosine, and tryptophan elevated, while glucose, sarcosine and 3-hydroxybutyrate decreased. The levels of metabolites in kidney, such as, leucine, valine, isoleucine and tyrosine, were increased, while taurine, glutamate, and glutamine decreased. The study provides several potential biomarkers for the toxicity mechanism research of RMP and shows that RMP may cause injury in kidney and liver and disturbance of several pathways, such as energy metabolism, oxidative stress, glucose and amino acids metabolism.

## Introduction

“Renqing Mangjue” pill (RMP) is one of the classical prescriptions of Traditional Tibetan Medicine (TTM), which was recorded in a Tibetan medical classic, the “The Four Medical Tantras” (Sibu Yidian, AD773–783; Zhao et al., 2010), and also officially approved by the Ministry of Health as one of the national protected Traditional Chinese Medicine (TCM) in 1997. RMP is well known for its superb therapeutic value. RMP has been commonly used for the treatment of digestive diseases, ulcerative colitis, peptic ulcer, herpes zoster, and food poisoning. However, with the increasingly popularity of Asian traditional medicine, concerns over the heavy metal presence aroused, whether it was added as ingredients, or caused contamination unintentionally (Aslam et al., 1979; Kang-Yum and Oransky, 1992; Hardy et al., 1995; Ernst, 2002; Saper et al., 2004). The safety of RMP is of great importance as high levels of heavy metal (such as, mercury, arsenic, and lead) in RMP have been detected (Zhao et al., 2010).

RMP utilizes as a complex herbal and mineral pharmacopeia. Cinnabar, which contains more than 96% (Ma et al., 2010) of mercuric sulfide (HgS) and heavy metal, is the main constituent of RMP that is a multi-ingredient formula used in variety conditions (Sallon et al., 2016). Up to now, no specific evidence has been documented the heavy metal poisoning, and the pharmacological effect of heavy metal in RMP is still a mystery. And less attention has been focused on the holistic metabolic changes of TTM exposure.

Metabolomics, as a systemic biology approach, has demonstrated great potential in many fields (Shockcor and Holmes, 2002), such as, toxicological evaluation (Arakaki et al., 2008), disease process (Oliver et al., 2002), and drug discovery (Lindon et al., 2003). Metabolomics provides an important method to trace the metabolic global changes in the biological processes in biofluids (e.g., blood and urine) or tissues (e.g., liver and kidney) of an organism (Nicholson et al., 2002; Xuan et al., 2011). Nuclear magnetic resonance (NMR) spectroscopy, with the advantages of rapid, non-destructive, and high-throughput, was used widely and frequently during the metabolic profiling (Shi et al., 2013). Additionally, NMR can be applied in biofluids (Semmar et al., 2014) or tissues (Graham et al., 2014) to generate metabolic profiles (Zhang et al., 2012). ^1^H NMR, which based on metabolomics, has been used in elucidate the toxicological mechanism of TCMs (Wang H. et al., 2013; Xu et al., 2013; Chen et al., 2016). Inductively coupled plasma mass spectrometry (ICP-MS), with the advantages of rapid and low detection limit, multi element analysis, and wide linear dynamic range, is considered as one of the most sensitive device for the determination of metal element in the biologic samples (Sarmiento-González et al., 2008).

In this work, ^1^H NMR-based metabolomics approach is employed to investigate the changes of metabolic profiles occurring in the plasma and tissues for the rat induced by different dosages of RMP. ICP-MS was also applied to determine the concentrations of heavy metals in plasma, liver and kidney tissue extracts. The purpose of this study is to comprehend the metabolic change of RMP on the rodent model based on the endogenous metabolism. Moreover, it is also hoped that the study will offer several potential toxicity information of RMP.

## Materials and methods

### Chemicals and reagents

RMP was purchased from Tso-Ngon Tibetan Medicine Hospital. Other chemicals were purchased from Sigma Chemical Co unless otherwise specified.

### Animal handling procedure and sampling

Animal experiments were conducted in accordance with the Guidelines for Animal Experimentation of Beijing University of Chinese Medicine, and the protocol was approved by the Animal Ethics Committee of the Institution. A total of 48 male Wistar rats (220 ± 10 g) purchased from Sibeifu Laboratory Animal Technology Co., Ltd. (Beijing, China; Rodent license No. SCXK 2011-0004). The animals were kept under controlled lighting (12 h light/dark cycle), temperature (22 ± 2°C), humidity (50 ± 10%) and were allowed for a week to acclimate environment prior to group allocation.

Rats were randomly allocated into four groups (*n* = 12) as following: low dose group (LD), middle dose group (MD), and high dose group (HD), which were administrated with RMP at a dose of 83.33, 333.33, and 1333.33 (mg/kg/day) respectively, and control group (NC) was treated with approximately equal volume of 0.9% saline solution. The intragastric administration was implemented per day for a consecutive 15 days. Body weights were recorded once a day. The doses of RMP for rats were equivalent to 5, 20, and 80 times of the normal clinical dosage.

The experiment last for 30 days. Half of all rats (*n* = 6 for each group) were euthanized on the 15th day (after the last administration of RMP), and the remaining rats were euthanized on the 30th day (15 days after the recovery period). Blood samples were collected and stored in a heparinized tube on ice. Half of the blood samples were separated and frozen for further ICP-MS analysis, and the plasma samples were obtained by centrifugation and stored at −80°C until further ^1^H NMR spectroscopic. The livers and kidneys were quickly removed from all rats at the time of death and were weighed up after rinsing with sterile 0.9% (w/v) sodium chloride solution. For liver and kidneys, half of tissues were immersed in 10% neutral-buffered formaldehyde for 24 h and embedded in paraffin to be stained with hematoxylin and eosin (H&E) for pathological analysis. And the residual tissues were placed in tubes and stored at −80°C until assessment.

### Sample preparation for NMR recording

The plasma samples were thawed only once in a biosafety fume hood, and then each 200 μL of plasma sample was mixed with 400 μL of deuterated phosphate buffer (NaH_2_PO_4_/K_2_HPO_4_, 0.045 mol/L, pH 7.47). The mixture was left to stand for a moment at room temperature and then centrifuged at 13,000 rpm for 15 min at 4°C for further purification. Aliquots of the supernatant (550 μL) of each sample were then transferred into a 5 mm NMR tube for NMR analysis (Feng et al., 2010).

Frozen kidney and liver tissues were homogenized in a mixture of an ice-cold extraction solvent (H_2_O: CH_3_CN 1:1) and centrifuged at 13,000 rpm at 4°C for 15 min. 1.00 mL volumes of supernatant (containing hydrophilic metabolites) was collected, concentrated under a stream of nitrogen and lyophilized and reconstituted in 600 μL 0.1 mol/L phosphate buffer (pH 7.47, 0.05% TSP, and 100% D2O), and 550 μL of the supernatant was decanted into a 5 mm NMR tube.

### ^1^H NMR spectroscopy

All ^1^H NMR spectra were acquired at a ^1^H frequency of 599.871 MHz using a Varian VNMRS 600 MHz NMR spectrometer, equipped with a 5-mm inverse-proton (HX) triple resonance probe. For plasma samples, the Carr–Purcell–Meiboom–Gill (CPMG) pulse sequence (RD-90°-(τ-180°-τ)_n_-ACQ) with a fixed total spin-spin relaxation delay 2 nτ of 320 ms was applied to observe the signals of micromolecules. Aqueous tissues extract sample spectra were recorded using One-dimensional RESAT pulse sequence, with a water presaturation suppression applied for a recycle delay of 2 s and a mixing time of 100 ms. For each sample, the free induction decays (FIDs) were measured with 128 scans producing 64 k data points over spectral width of 12,019 Hz. Before Fourier transformation, the FIDs were zero-filled to 64 k points and processed with 0.5 Hz exponential line-broadenings (Jianxin et al., 2016). Standard two-dimensional (2D) NMR experiments such as ^1^H-^1^H COSY, ^1^H-^13^C HSQC and ^1^H-^13^C HMBC were also acquired to assist metabolite assignment.

### ICP-MS measurement of blood, kidney and liver extracts

The kidney and liver tissues were freeze-dried for 24 h at −80°C and homogenized individually with agate mortar prior to analysis.

Two tracer elements (^75^As, ^202^Hg) in all samples (Blood, kidney and liver) were quantified by ICP-MS (Agilent Technologies, 7500 Ce) after a modified acid digestion with HNO_3_+H_2_O_2_ (5:1) by microwave assisted digestion (CEM Co., MARS). The mercury and arsenic standard stock solution of 100 mg/L were purchased to measure the calibration curves, and were diluted to the standard solutions with 5% (w/w) HNO_3_ in ultrapure deionized water. The external calibration procedures were employed for the quantification of the samples (Khan et al., 2015). Quantitation was achieved by a four-point calibration curve with a series of concentrations (0.1–100 ng mL^−1^), using ^72^Ge as internal standards for As and ^209^Bi for Hg.

### Data analysis

The ^1^H NMR spectra was processed using MestReNova software (Version 7.1.0, Mestrelab, Inc.), and the spectra was manually baseline- and phase-corrected and referenced to the TSP signal (δ0.00). The plasma spectra were referenced to resonance of lactate at δ1.33, and the tissues spectra were referenced to the chemical shift of TSP (δ0.00). The spectral region δ9.0–0.5 for each plasma sample and the spectral region δ9.5–0.5 for each tissue sample were all automatically data reduced to integrated segments of equal width (0.002 ppm). Spectral regions (δ5.20–4.70) were excluded to eliminate variations caused by imperfect water suppression. The integrated data of the remaining bins were normalized to the total sum of integrals for each spectrum to compensate for the effect of variation (Feng et al., 2011).

The NMR spectral data sets were examined with SIMCA-P+12.0 (Umetrics, Sweden) for multivariate statistical analysis. Principal component analysis (PCA) was usually implemented with mean-centered NMR data to identify general trends and outliers and to examine group clustering. Then, the supervised partial least squares discriminant analysis (PLS-DA) and orthogonal projections to the latentstructures discriminant analysis (OPLS-DA) at a unit variance-scaled approach were performed to specify the metabolic variations associated with the drug. The parameters of R2 and Q2 were used to assess the goodness of fitness and the predictive ability of the models, respectively. In order to reflect the variables contributing to the classification, the color-coded loadings plots with absolute value of coefficients (*r*) were used to identify significantly altered metabolites, and were generated with MATLAB R2012a (http://www.mathworks.com) with some in-house modifications (Jianxin et al., 2016).

In order to discriminate the variables contributing to sample clustering among the groups, the variable importance in the projection (VIP) values of all peaks from OPLS-DA models was analyzed. Moreover, an independent sample *t*-test was imported into detecting significant differences between the two groups by SPSS Statistics Base 17.0 (SPSS Inc., USA). In this study, we used an a priori cutoff VIP value > 1 of multivariate and *p* < 0.05 of univariate statistical significance to identify the distinguishing metabolites.

## Results

### Histopathology

**Figure 1:**
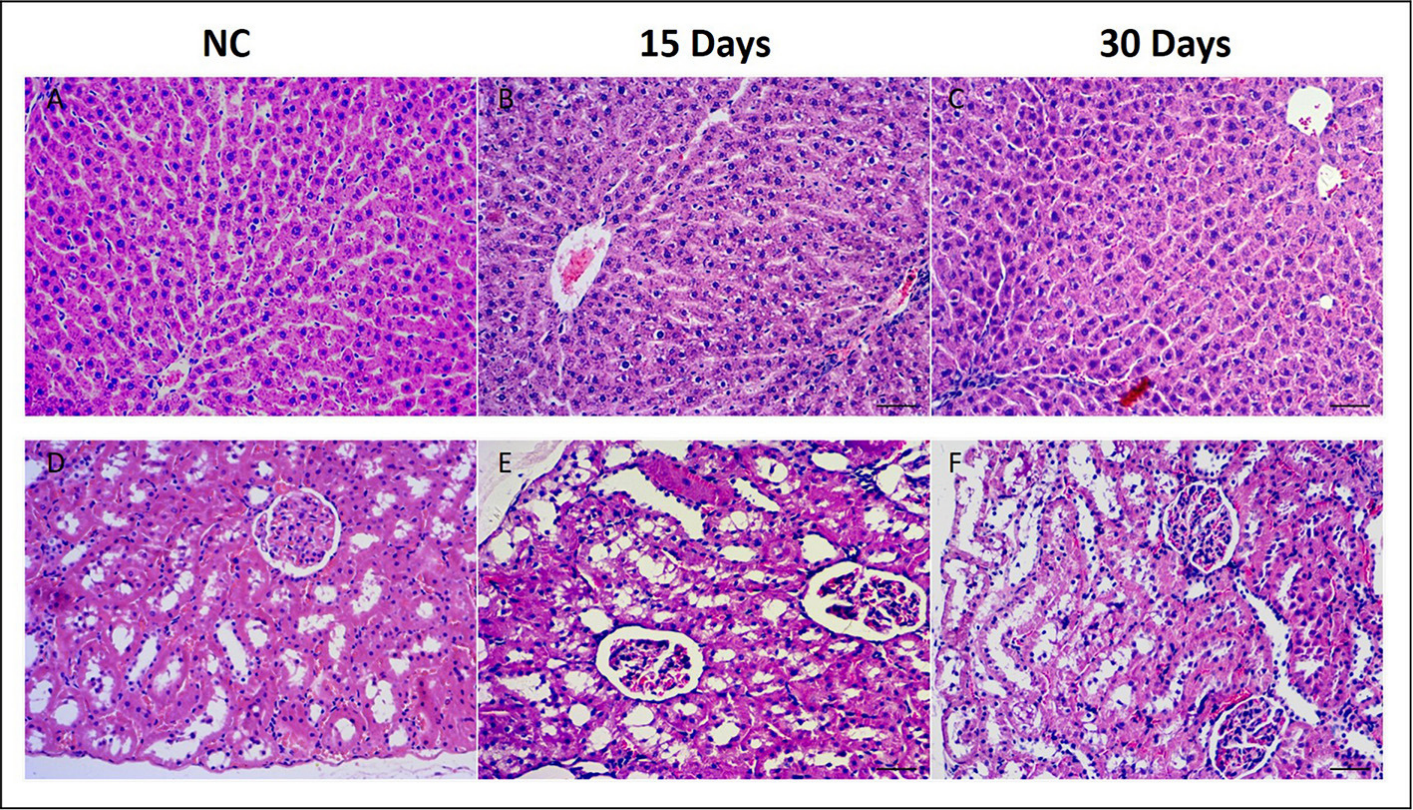
Histopathological photomicrographs of control group **(A)** and high does group **(B,C)** liver tissues, control group **(D)** and high does group **(E,F)** kidney tissues by HE staining (10x).

The histopathological changes in the liver and kidney were examined after HE staining. Compare to the NC group (Figure 1A), liver of HD rats on 15th days showed hydropic degeneration, inflammatory and severe epithelial necrosis (Figure 1B). And the pathologic injuries alleviated in liver tissue (Figure 1C) on 30th days. Kidney of the NC rat showed an normal structure in renal glomerulus and tubule (Figure 1D), while HD rats on 15th days showed slight pathologic transformations of renal glomerulus and tubule, such as, tubular epithelial cell degeneration and diaphanous tubular cast (Figure 1E). Fifteen days after regeneration phase showed markedly recovery trends for kidney injuries which induced by RMP (Figure 1F).

### ^1^H NMR spectra of plasma and tissue extracts sample

**Figure 2:**
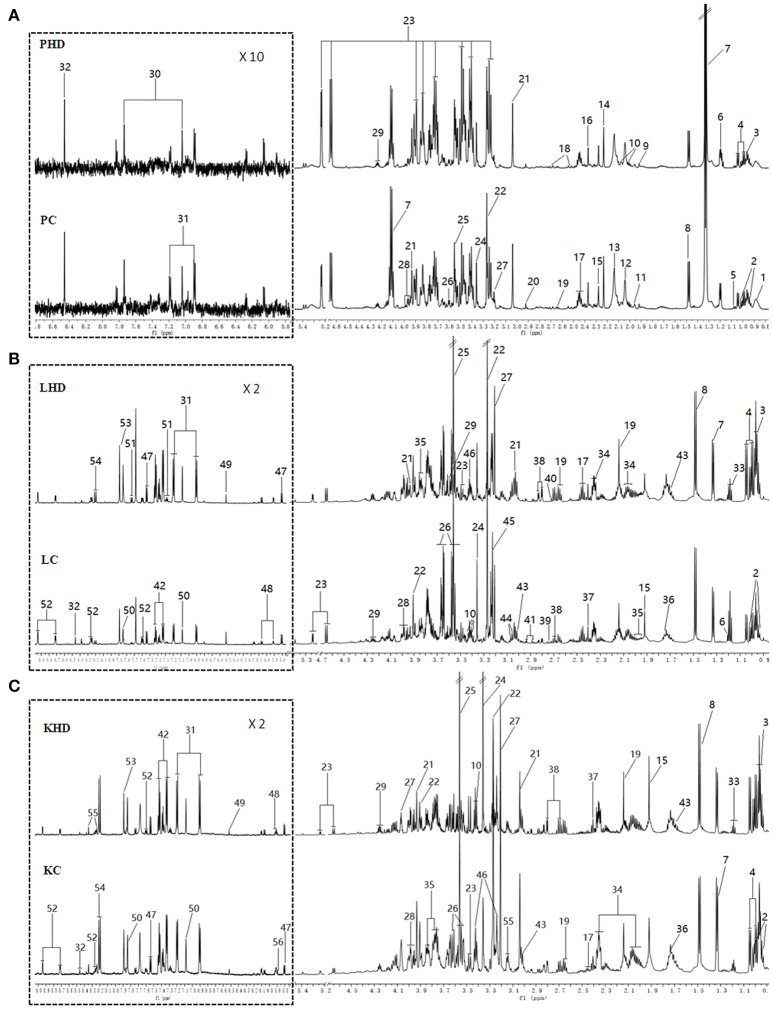
Typical 600 MHz ^1^H NMR spectra from plasma **(A)**, aqueous liver **(B)** and kidney **(C)** extracts. (PHD, LHD, and KHD) plasma, liver extract, and kidney extract from a rat dosed with high levels of RMP; (PC, LC, and KC) plasma, liver extract, and kidney extract from a control rat. The aromatic regions of plasma spectra are magnified 10 times compared to those of corresponding aliphatic regions. The aromatic regions of kidney and liver spectra are magnified 2 times compared to the aliphatic regions. Distinguished metabolites: 1, LDL; 2, isoleucine; 3, leucine; 4, valine; 5, isobutyrate; 6, 3-Hydroxybutyrate; 7, lactate; 8, alanine; 9, acetate; 10, proline; 11, CH2CH2C = C lipid; 12, NAC1; 13, NAC2; 14, acetone; 15, acetoacetate; 16, pyruvate; 17, glutamine; 18, citrate; 19, methionine; 20, N,N-dimethylglycine; 21, creatine; 22, betaine; 23, glucose; 24, glycerophosphoylcholine; 25, glycine; 26,glycerol; 27,choline; 28, serine; 29, threonine; 30, methylhistidine; 31,tyrosine; 32, formate; 33, ethanol; 34, glutamate; 35, homoserine; 36, arginine; 37, succinate; 38, aspartate; 39, sarcosine; 40, dimethylamine; 41, asparagine; 42, phenylalanine; 43, lysine; 44, ornithine; 45, phosphocholine; 46, taurine; 47, uracil; 48, cytidine; 49, fumarate; 50, histidine; 51, tryptophan; 52, niacinamide; 53, xanthine; 54, hypoxanthine; 55, inosine; 56, uridine; 57, urea.

Representative 600 MHz ^1^H NMR spectra of plasma and tissue extracts obtained from control and HD on 15th days were showed in Figure 2, with major metabolites in the integrated regions assigned. NMR spectral data was analyzed and specific metabolites were identified by the previously reported reference (Kim et al., 2014; Guo et al., 2015; Liao et al., 2016; Li et al., 2016) and in-house NMR database and the database of Chenomx NMR Suite 6.0 (Chenomx, Edmonton, Canada). Metabolites detected in plasma, tissue extracts including amino acids, glycolysis products, tricarboxylic acid cycle (TCA cycle) intermediates and choline metabolites, etc.

### Metabolic responses of plasma after RMP administration

**Figure 3:**
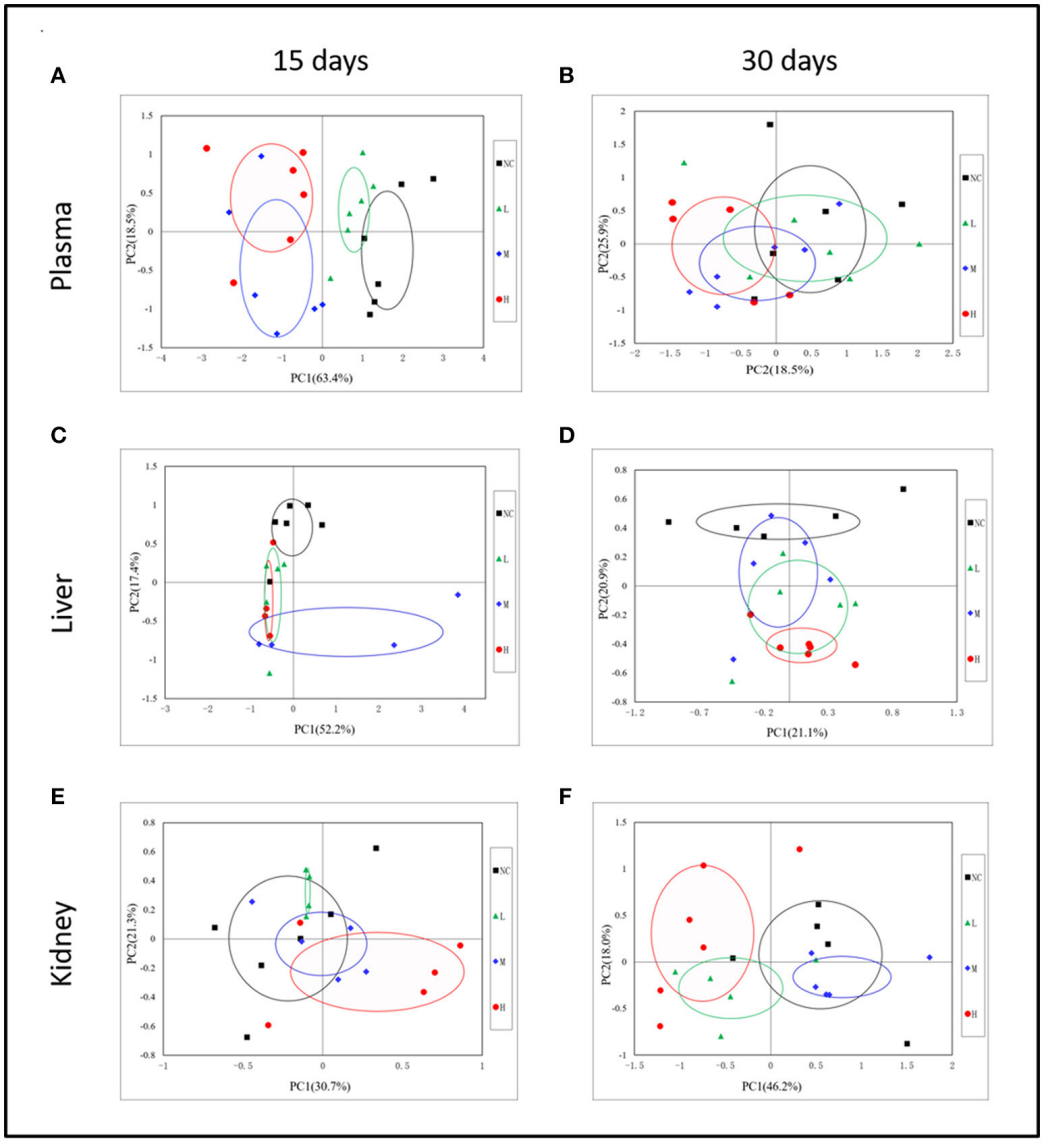
Representative PCA score plots (PC1 vs. PC2) derived from the ^1^H NMR data of plasma **(A,B)**, liver extract **(C,D)**, and kidney extract **(E,F)** from control and dosed groups at day 15 and day 30.

In order to display the overall metabolic trends and find the possible outliers, the NMR data of all samples was subjected to PCA analysis. The PCA score plots (Figure 3A) of plasma ^1^H NMR spectra 15 days after administration with RMP displayed evident differentiation between NC and dosed groups along the first principal component, except a partial overlap between NC group and LD group. As for withdrawal period (Figure 3B), the discrimination between control and RMP groups was less clear compared with 15 days before. PLS-DA on 15th day (Figure S1A) highlighted the distinctive separations between controls and RMP groups but the overlapping between MD and HD groups still existed, which implied the similar metabolic phenotype between MD and HD groups in plasma, while the separation was less clear on 30th day (Figure S1B).

**Figure 4:**
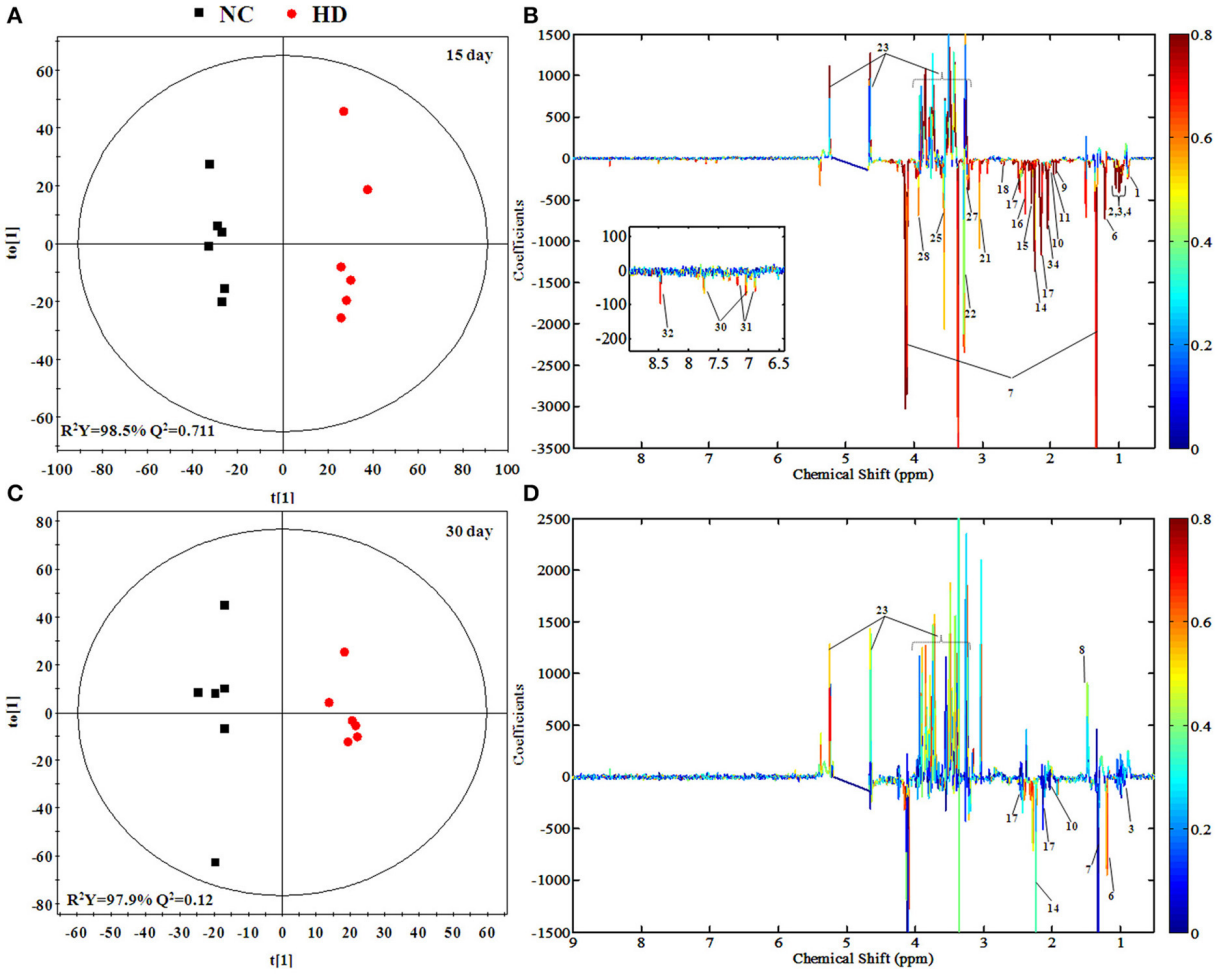
OPLS-DA scores plots **(A,C)** and coefficient loading plots **(B,D)** derived from ^1^H NMR spectra of plasma from HD and NC group at day 15 and day 30. The color code corresponds to the correlation coefficients of the metabolic variables. The loading plots identifying discriminatory metabolites between HD and NC group are based on the first principal component [*t*_(1)_]. Signals with a positive direction relate to the abundance of metabolites in the groups in the positive direction of [t_(1)_], and vice versa.

To screen metabolomics differences between control group and RMP groups and compare the significance of the identified metabolites contribution during the physiological alterations, pairwise OPLS-DA score plots and corresponding loading plots of plasma samples were performed in Figure 4 and Figure S2. The OPLS-DA score plots gave a clear separation between RMP groups and NC group. Color-coded coefficient plots showed detailed metabolites changes induced by RMP. Metabolites exhibiting significant changes (*p* < 0.05) were recognized as potential biomarkers according to the absolute cutoff value of correlation coefficients (|*r*|) and VIP value were summarized in Tables 1, 2. Results suggest little perturbation in low RPM group after 15-day oral administration. However, compared with control group, many metabolites in high dose RMP group, such as, glucose, betaine, and creatine, remarkable increased, while lipids, 3-hydroxybutate, pyruvate, citrate, branched-chain amino acids (BCAAs; valine, leucine, and isoleucine), glutamate and glutamine decreased at the same time. Most of the disturbed metabolites were ameliorated after the withdrawal of RMP, but there were still some significant metabolites in HD, including alanine, acetone, lactate, proline, glucose, and betaine.

**Table 1:** Significant change of the metabolites derived from the NMR data from different group at day 15.

	**Metabolites**		**L–C**		**M–C**		**H–C**	
	**Compound name**	**Chemical shift**	**Fold^a^**	**VIP**	**Fold**	**VIP**	**Fold**	**VIP**
Plasma	LDL/VLDL	**0.87(m)**	0.99	0.32	0.79	1.03	0.60^**^^b^	1.34
	Isoleucine	**0.95(t)**, 1.03(d), 1.46(m)	0.93	0.99	0.73^*^	1.47	0.64^**^	1.48
	Leucine	0.96(d), **0.97(d)**, 1.70(m), 3.72(m)	0.88	1.18	0.67^**^	1.64	0.67^**^	1.39
	Valine	**0.99(d)**, 1.04(d)	0.93	0.80	0.71^**^	1.56	0.66^*^	1.36
	3-Hydroxybutyrate	**1.21(d)**, 2.30(dd), 2.40(dd)	0.98	0.06	0.57^*^	1.46	0.43^**^	1.41
	Acetate	**1.92(s)**	0.77^*^	1.40	0.71^**^	1.59	0.60^**^	1.39
	Lipids	**1.97(m)**	0.82^**^	1.66	0.76^**^	1.61	0.63^**^	1.50
	Proline	2.01(m), **2.08(m)**, 2.35(m), 4.12(m)	0.87^*^	1.39	0.78^**^	1.61	0.72^**^	1.56
	Acetone	**2.24(s)**	0.88	0.51	0.46^**^	1.79	0.44^**^	1.33
	Glutamate	2.05(m), **2.34(m)**, 3.76(t)	0.92	0.99	0.79^**^	1.56	0.72^**^	1.31
	Pyruvate	**2.37(s)**	0.96	0.21	0.81	1.15	0.69^*^	1.05
	Glutamine	2.14(m), **2.45(m)**, 3.76(t)	0.87^*^	1.32	0.85^*^	1.29	0.72^*^	1.22
	Citrate	2.54(AB), **2.69(AB)**	0.84^*^	1.43	0.73^*^	1.33	0.61^**^	1.47
	Glucose	**3.5-4.0(m)**, 4.65(d), 5.23(d)	1.14^*^	1.46	1.35^*^	1.39	1.58^**^	1.63
	Glycine	**3.56(s)**	0.80	1.12	0.76^**^	1.56	0.70^*^	1.13
	Betaine	3.26(s), **3.91(s)**	1.01	0.34	1.23^*^	1.32	1.24^**^	1.36
	Creatine	3.03(s), **3.93(s)**	0.94	0.54	1.12^*^	1.57	1.16^*^	1.68
	Serine	**3.95(m)**	0.88	1.14	0.80^*^	1.59	0.72^*^	1.29
	Choline	**3.20(s)**, 3.51(m), 4.05(m)	0.88	0.82	0.73	0.71	1.03^*^	1.22
Liver	Leucine	0.96(d), **0.97(d)**, 1.70(m), 3.72(m)	1.31^*^	1.16	1.25	1.21	1.62^**^	1.75
	Valine	**0.99(d)**, 1.04(d)	1.24^*^	1.12	1.09	1.09	1.47^**^	1.57
	3-Hydroxybutyrate	**1.21(d)**, 2.30(dd), 2.40(dd)	1.02	0.56	0.40^**^	1.41	0.57^**^	1.60
	Isoleucine	0.95(t), **1.03(d)**, 1.46(m),	1.06	1.00	0.59^*^	1.31	0.80^**^	1.43
	Alanine	**1.48(d)**, 3.81(q)	1.20	1.14	1.02	0.98	1.26^**^	1.40
	Glutamine	2.14(m), **2.46(m)**, 3.76(t)	0.81^**^	1.88	0.55^**^	1.35	0.74^**^	1.77
	Sarcosine	**2.74(s)**, 3.61(s)	1.02	0.38	0.61^*^	1.35	0.80^**^	1.49
	Lysine	1.46(m), 1.72(m), 1.90(m), **3.03(t)**	1.13	1.16	1.01^*^	1.00	1.34^**^	1.68
	Glucose	**3.5-4.0(m)**, 4.65(d), 5.23(d)	1.10	0.89	0.84^*^	1.36	0.84^*^	1.35
	Lactate	1.32(d), **4.11(q)**	0.90	1.32	0.81^*^	1.22	0.80^**^	1.44
	Proline	2.01(m), 2.08(m), 2.35(m), **4.12(m)**	1.11	0.79	1.00	1.00	1.19^**^	1.53
	Tyrosine	**6.90(d)**, 7.19(d)	1.10	1.21	1.08	1.09	1.47^**^	1.69
	Phenylalanine	**7.32(m)**, 7.37(m), 7.41(m)	0.97	1.19	0.85^*^	1.24	0.59^*^	1.72
	Tryptophan	7.29(t), 7.33(s), 7.55(d), **7.74(d)**	1.38	0.93	1.05	0.90	1.51^*^	1.33
Kidney	Leucine	0.96(d), **0.97(d)**, 1.70(m), 3.72(m)	1.12^*^	1.39	1.20^*^	1.69	1.21^*^	1.71
	Valine	**0.99(d)**, 1.04(d)	1.06	0.84	1.14^*^	1.72	1.15^*^	1.55
	Isoleucine	0.95(t), **1.03(d)**, 1.46(m)	1.09	1.18	1.28^*^	1.59	1.38^*^	1.88
	3-Hydroxybutyrate	**1.21(d)**, 2.30(dd), 2.40(dd)	0.88	0.49	0.82	0.59	0.61^*^	1.44
	Alanine	**1.48(d)**, 3.81(q)	1.01	0.27	1.21^**^	1.31	1.25^**^	1.68
	Glutamate	**2.05(m)**, 2.34(m), 3.76(t)	1.06	0.99	1.08^*^	1.28	1.08^*^	1.30
	Dimethylamine	**2.72(s)**	0.98	0.86	0.88^*^	1.43	0.88^**^	1.44
	Phenylalanine	7.32(m), 7.37(m), **7.41(m)**	0.98	1.05	0.90	1.40	0.83^**^	1.70
	Taurine	**3.25(t)**, 3.42(t)	0.81^*^	1.47	0.66^**^	1.73	0.68^**^	1.77
	Glycerol	3.55(dd), **3.64(dd)**, 3.78(m)	0.84	0.98	0.76^*^	1.16	0.68^**^	1.43
	Proline	2.01(m), 2.08(m), 2.35(m), **4.12(m)**	1.01	0.62	1.03	0.81	1.09^*^	1.32
	Glucose	3.5-4.0(m), 4.65(d), **5.23(d)**	0.76	1.05	1.11	0.41	1.44^*^	1.44
	Tyrosine	6.90(d), **7.19(d)**	1.18^*^	1.38	1.22^**^	1.55	1.26^**^	1.59

a *Fold change values, color coded according to log_2_(fold), red the increased and blue the decreased in each groups. Color bar.* 
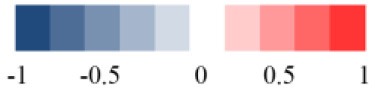

b *The p-values were obtained from student's t-test. The chemical shifts in boldface were that we used in calculating integrals and p-values*.

* *p < 0.05*,

** p < 0.01.

**Table 2:** Significant change of the metabolites derived from the NMR data from different group at day 30.

	**Metabolites**		**L–C**		**M–C**		**H–C**	
	**Compound name**	**Chemical shift**	**Fold^a^**	**VIP**	**Fold**	**VIP**	**Fold**	**VIP**
Plamsa	Leucine	0.96(d), **0.97(d)**, 1.70(m), 3.72(m)	0.88	0.10	0.67	0.31	0.67^*^^b^	1.82
	Lactate	**1.32(d)**, 4.11(q)	0.86	0.08	0.58	1.28	0.55^*^	1.80
	Alanine	**1.48(d)**, 3.81(q)	1.12	1.60	0.87	1.55	0.92^*^	2.03
	Proline	**2.01(m)**, 2.08(m), 2.35(m), 4.12(m)	0.99	1.77	0.76	1.11	0.80^**^	2.10
	Acetone	**2.24(s)**	0.91	1.03	0.44	1.54	0.61^*^	1.90
	Betaine	3.26(s), **3.91(s)**	0.92	0.08	0.97	1.35	0.95^*^	2.01
	Glycine	**3.56(s)**	0.80	0.51	0.70	0.64	0.76^*^	2.00
	Glutamine	2.14(m), 2.45(m), **3.76(t)**	1.13	0.75	1.44	1.10	1.31^*^	1.94
	Glucose	3.5-4.0(m), 4.65(d), **5.23(d)**	1.07	0.56	1.42	1.17	1.41^*^	2.04
Liver	Isoleucine	**0.95(t)**, 1.03(d), 1.46(m), 3.66(d)	1.04	1.02	1.04	1.03	1.10^*^	1.55
	3-Hydroxybutyrate	**1.21(d)**, 2.30(dd), 2.40(dd)	0.87	0.57	0.76	1.57	0.70^*^	1.51
	Valine	0.99(d), **1.04(d)**	1.09	1.05	1.05	0.99	1.11^*^	1.49
	Lysine	1.46(m), 1.72(m), 1.90(m), **3.03(t)**	1.09	1.03	1.06	1.00	1.12^**^	1.81
	Betaine	**3.26(s)**, 3.91(s)	0.84^*^	2.12	0.82^*^	1.44	0.68^**^	1.92
	Glucose	**3.5-4.0(m)**, 4.65(d), 5.23(d)	0.91^*^	1.53	0.81^**^	1.67	0.86^**^	1.98
	Creatine	3.03(s), **3.93(s)**	1.38	1.47	1.12^*^	0.96	1.11^**^	1.70
	Lactate	1.32(d), **4.11(q)**	1.19	1.71	1.04	0.94	1.05^*^	1.39
	Proline	2.01(m), 2.08(m), 2.35(m), **4.12(m)**	1.13	1.34	1.02	0.74	1.11^**^	1.86
	Phenylalanine	**7.32(m)**, 7.37(m), 7.41(m)	0.95	0.65	0.92	0.95	1.02^*^	1.56
Kidney	3-Hydroxybutyrate	**1.21(d)**, 2.30(dd), 2.40(dd	1.01	0.19	0.92	0.63	0.68^**^	1.60
	Glutamate	**2.05(m)**, 2.34(m), 3.76(t)	1.07	1.01	1.05	0.88	1.15^*^	1.45
	Succinate	**2.41(s)**	0.93	1.37	1.15	1.40	1.24^**^	1.70
	Taurine	3.25(t), **3.42(t)**	0.99	0.44	0.98	0.41	0.90^*^	1.30
	Glycine	**3.56(s)**	1.07	0.69	1.09	1.09	1.13^*^	1.37
	Serine	**3.95(m)**	1.00	0.06	1.00	0.22	1.08^*^	1.46
	Proline	2.01(m), 2.08(m), 2.35(m), **4.12(m)**	1.01	0.19	1.01	0.25	1.12^**^	1.72
	Glucose	3.5-4.0(m), **4.65(d)**, 5.23(d)	0.98	0.95	0.98	0.51	0.80^*^	1.37
	Tyrosine	**6.90(d)**, 7.19(d)	0.95	0.71	0.87	1.41	1.22^*^	1.51

a *Fold change values, color coded according to log_2_(fold), red the increased and blue the decreased in each groups. Color bar.* 
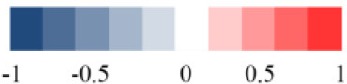

b *The p-values were obtained from student's t-test. The chemical shifts in boldface were that we used in calculating integrals and p-values*.

* *p < 0.05*,

** p < 0.01.

### Metabolic responses of liver after RMP administration

Some separation of the liver samples from control and RMP groups were evident in PCA plot on 15th day (Figure 3C), but complete separation among RMP groups were not achieved. 52.2% of the variance was explained by PC1, while 17.4% was explained by PC2. It was observed that there was still a discrimination among the control and RMP groups on 30th day in PCA plot (Figure 3D), especially between the NC and HD group. The score plot was obtained with the first two PCs presenting 21.2 and 20.9% variance, respectively. The PLS-DA score plot of liver on 15th day showed in Figure S1C, and four groups displayed a distinct separation. While on day 30 (Figure S1D) the separation was less obvious.

**Figure 5:**
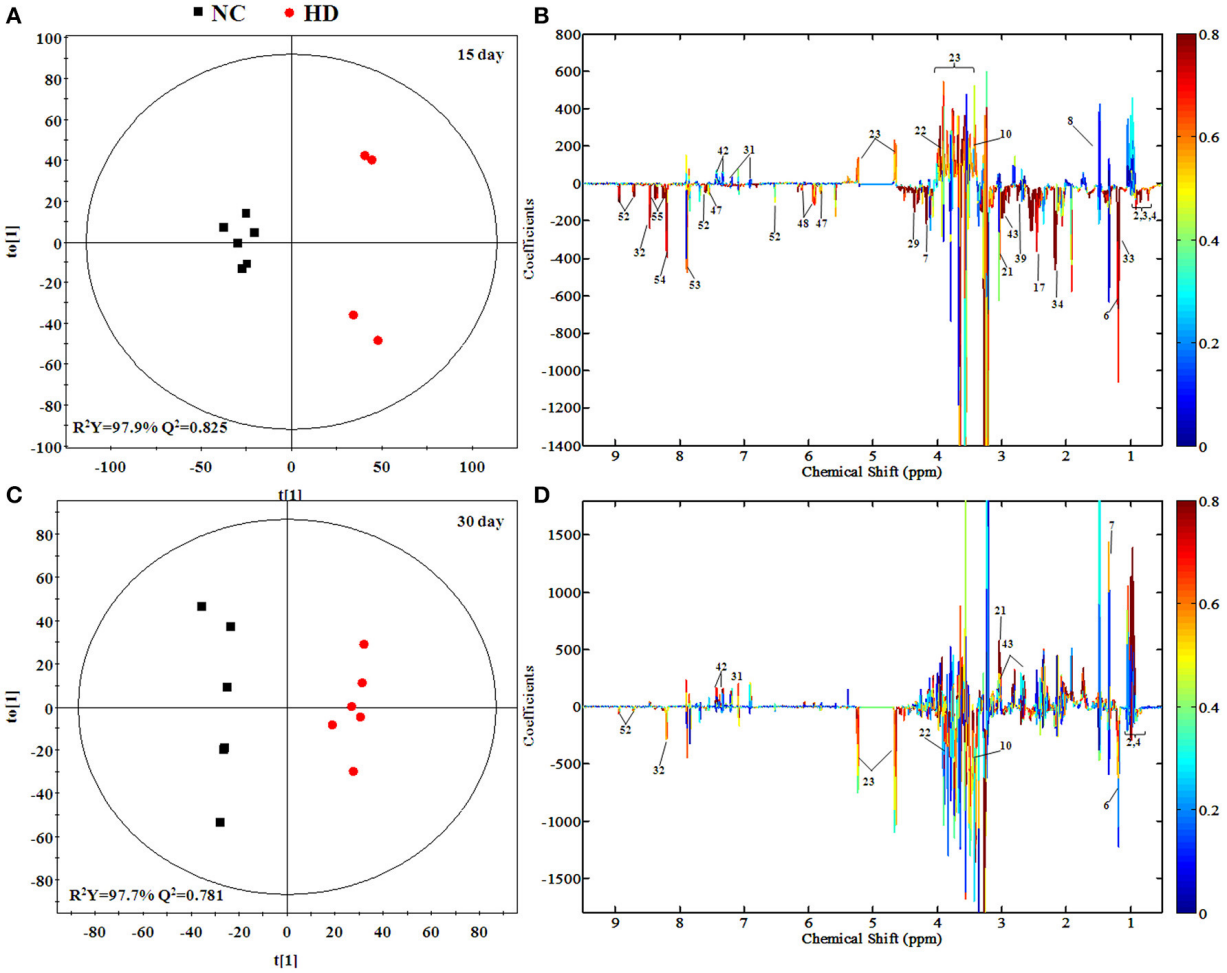
OPLS-DA scores plots **(A,C)** and coefficient loading plots **(B,D)** derived from ^1^H NMR spectra of liver extract from HD and NC group at day 15 and day 30. The color code corresponds to the correlation coefficients of the metabolic variables. The loading plots identifying discriminatory metabolites between HD and NC group are based on the first principal component [*t*_(1)_]. Signals with a positive direction relate to the abundance of metabolites in the groups in the positive direction of [*t*_(1)_], and vice versa.

Figure 5 and Figure S3 showed the OPLS-DA score plots and corresponding loading plots of liver samples. The ^1^H NMR-identified relative metabolites in liver samples of dose groups are shown in Tables 1, 2. The metabolomics analysis of liver showed elevated concentrations of valine, leucine, proline, tyrosine, and tryptophan, as well as decreasing glucose, sarcosine and 3-hydroxybutyrate from RMP-administrated rats. The difference between control group and RMP groups became less significant on 15th day after the last administration. Metabolites regulation of the RMP group could be observed, which included leucine, valine, lactate, proline, glucose, and betaine.

### Metabolic responses of kidney after RMP administration

According to the PCA score plots of the kidney tissue in Figure 3E, slight separation was observed on 15th day, with partial overlap between RMP groups and control group. However, the differences between control and RMP groups were observed on 30th say in PCA plot (Figure 3F), indicating that the toxicity of RMP in kidney may delay. From the PLS-DA score plot, the differentiation of RMP groups and control group is observed on 15th day (Figure S1E), while the separation was less apparent on 30th day (Figure S1F).

**Figure 6:**
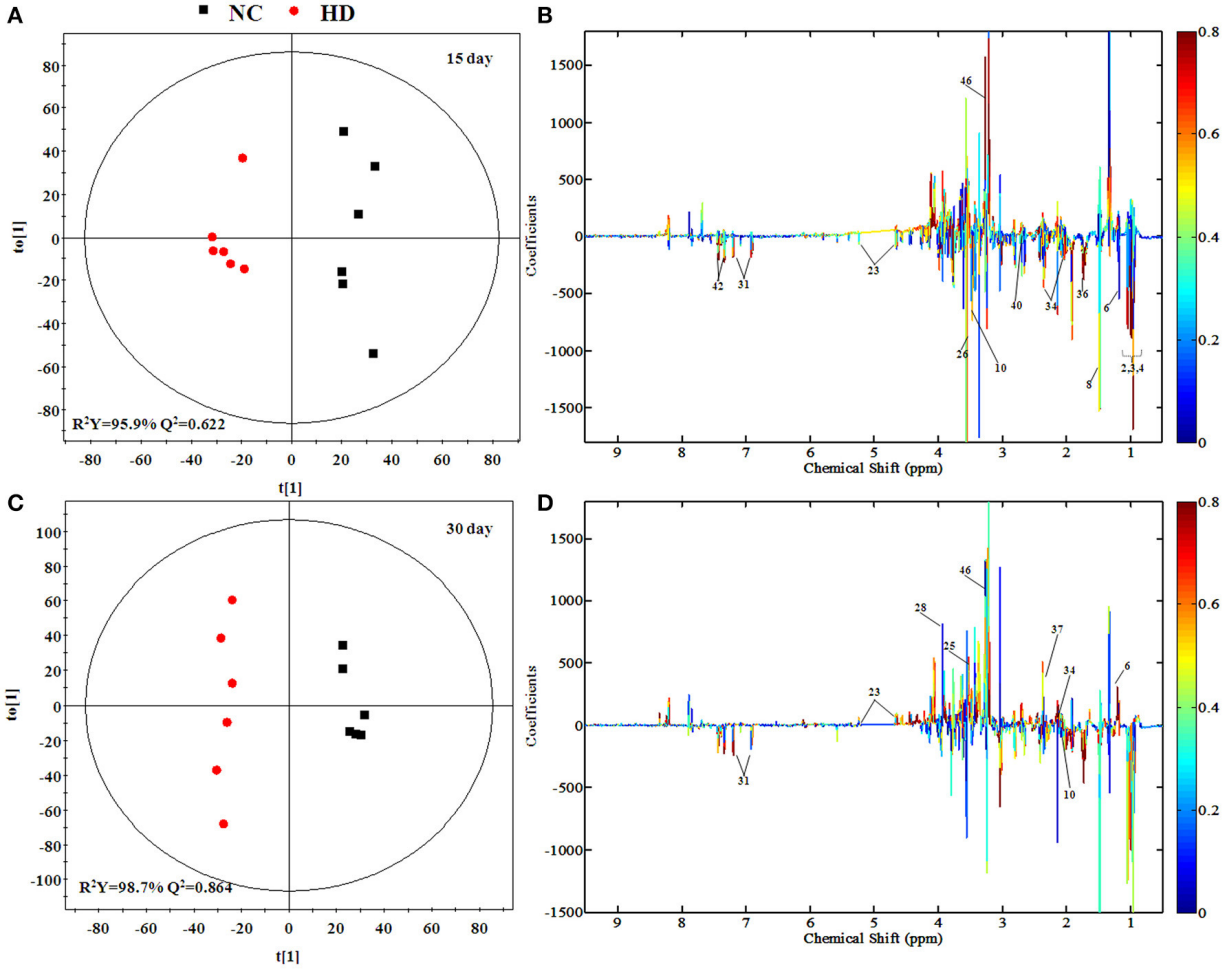
OPLS-DA scores plots **(A,C)** and coefficient loading plots **(B,D)** derived from ^1^H NMR spectra of kidney extract from HD and NC group at day 15 and day 30. The color code corresponds to the correlation coefficients of the metabolic variables. The loading plots identifying discriminatory metabolites between HD and NC group are based on the first principal component [*t*_(1)_]. Signals with a positive direction relate to the abundance of metabolites in the groups in the positive direction of [*t*(1)], and vice versa.

The separation between NC and each RMP group is exhibited in the score plots of OPLS-DA and corresponding loading plots of kidney samples were performed (Figure 6 and Figure S4). The color-coded co-efficient plots disclosed the increased levels of leucine, valine, isoleucine, and tyrosine, together with the decreased levels of taurine, betaine, choline, 3-hydroxybutyrate, and no evident recovery was displayed on 30th day. The identification of significant class-discriminating metabolites was summarized in Tables 1, 2.

### Metabolic pathway analysis

**Figure 7:**
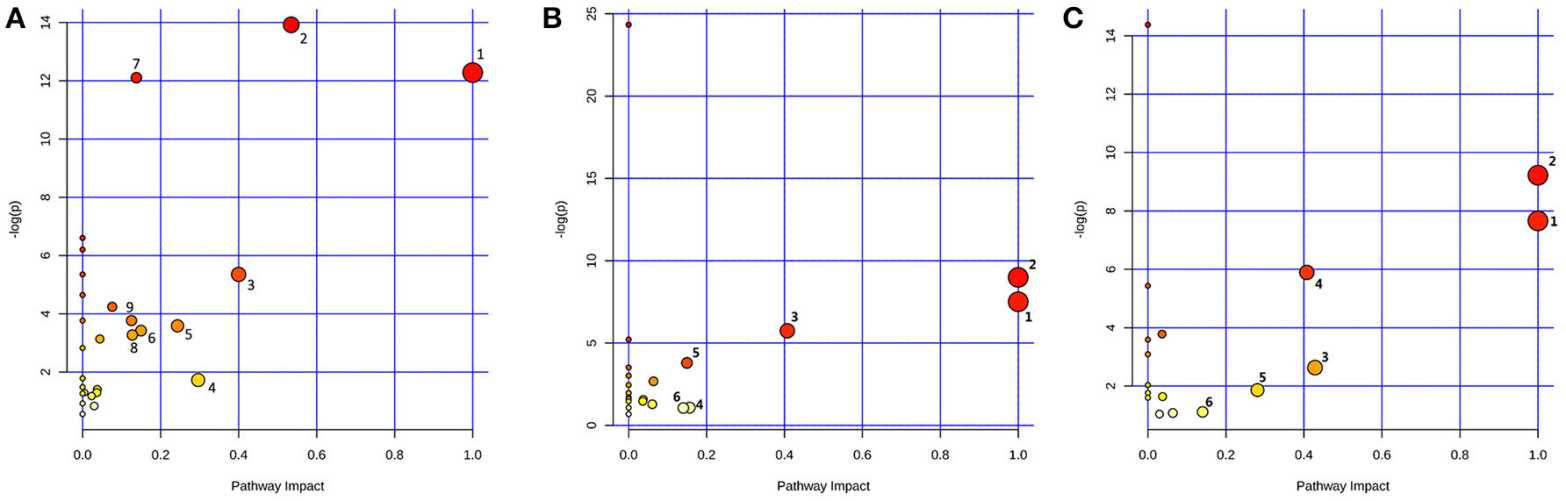
Metabolic Pathway Analysis of plasma **(A)**, liver **(B)**, and kidney **(C)**. The impact is the pathway impact value calculated from pathway topology analysis. Plasma: 1.Valine, leucine, and isoleucine biosynthesis; 2.Glycine, serine and threonine metabolism; 3.Methane metabolism; 4.Glyoxylate and dicarboxylate metabolism; 5.Pyruvate metabolism; 6.Alanine, aspartate and glutamate metabolism; 7.Aminoacyl-tRNA biosynthesis; 8.Glycolysis or Gluconeogenesis; 9.Citrate cycle (TCA cycle). Liver: 1.Phenylalanine, tyrosine and tryptophan biosynthesis; 2.Valine, leucine and isoleucine biosynthesis; 3.Phenylalanine metabolism; 4.Tryptophan metabolism; 5.Alanine, aspartate and glutamate metabolism; 6.Tyrosine metabolism. Kidney: 1.Phenylalanine, tyrosine and tryptophan biosynthesis; 2.Valine, leucine and isoleucine biosynthesis; 3.Taurine and hypotaurine metabolism; 4.Phenylalanine metabolism; 5.Glycerolipid metabolism; 6.Tyrosine metabolism.

Based on the identified biomarkers, MetaboAnalyst 3.0 (http://www.metaboanalyst.ca/MetaboAnalyst/) was performed to identify the relevant pathways involved in the study conditions. According to previous literature, pathways with the impact value above 0.1, which is calculated from pathway topology analysis, were screened out as potential target pathway. Finally, there were 9 potential target pathways identified in plasma samples, including valine, leucine and isoleucine biosynthesis, glycine, serine and threonine metabolism, methane metabolism, glyoxylate and dicarboxylate metabolism, pyruvate metabolism, alanine, aspartate and glutamate metabolism, aminoacyl-tRNA biosynthesis, glycolysis or gluconeogenesis and citrate cycle (TCA cycle) (Figure 7A). And there were 6 potential target pathways identified in liver (Figure 7B), including phenylalanine, tyrosine and tryptophan biosynthesis, valine, leucine and isoleucine biosynthesis, phenylalanine metabolism, tryptophan metabolism, alanine, aspartate and glutamate metabolism and tyrosine metabolism. There were 6 potential target pathways including Phenylalanine, tyrosine and tryptophan biosynthesis, valine, leucine and isoleucine biosynthesis, taurine and hypotaurine metabolism, phenylalanine metabolism, glycerolipid metabolism and tyrosine metabolism were identified in kidney samples (Figure 7C). The details of pathways were displayed in Tables S1–S3, Supporting Information.

### ICP-MS determination of As and Hg in plasma and tissues samples

**Figure 8:**
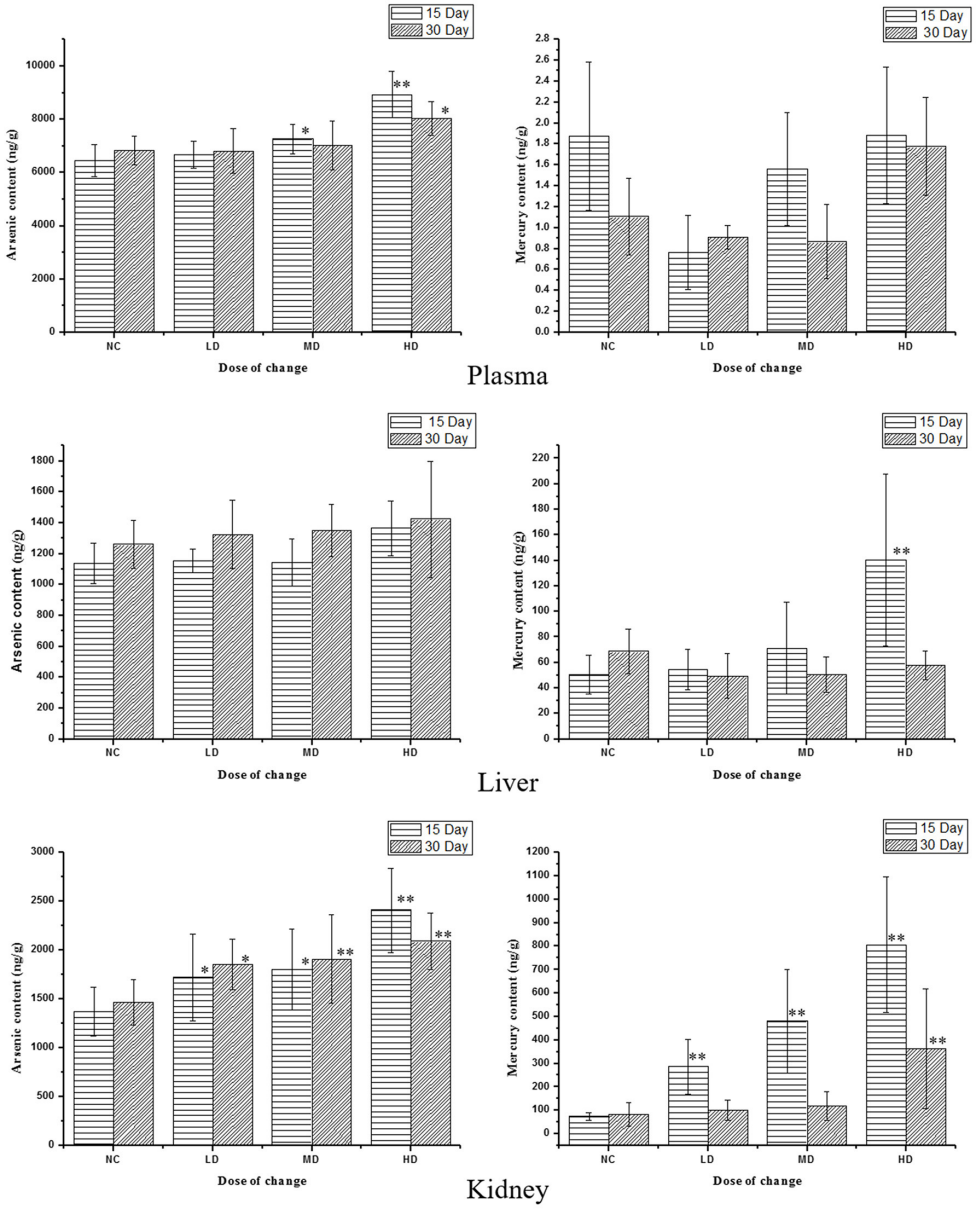
Content of As and Hg in rats serum and tissue samples on various doses. ^*^p < 0.05, ^**^p < 0.01 vs. control group.

RMP contains a great deal of mineral element including Hg and As (Zeng-cai-dan et al., 2016), which were besides the human body essential trace elements and may affect the metabolism in certain way. So it was necessary to measure the concentrations of heavy metal in tissues and biofluid. The results were summarized in Table S4 and Figure 8. The levels of As and Hg in rats plasma, liver and kidney tissues were dose-dependent. RMP groups, especially HD group, showed an increase of As concentrations after 15-day oral administration, and the concentration of As in plasma and kidney declined 15 days after the last administration, while the concentration of As showed a slightly elevation in liver. The concentration of Hg showed the same trend after the RMP administration. It was shown in Figure 8 that the Hg level in kidney was substantially higher than that in plasma and liver for all group. The level of Hg in kidney was substantially higher than that in plasma and liver of all RMP groups and even in the control group, which means that the accumulation of Hg was apparent in the kidney. Nevertheless, during the regeneration phase, a remarkable decrease of Hg was observed in both plasma and tissue samples.

## Discussion

The toxic effect of RMP was firstly evaluated by pathological section. Pathology results of kidney showed slight pathologic changes of renal glomerulus and tubule, such as, tubular epithelial cell degeneration and diaphanous tubular cast. Pathology results of liver showed hydropic degeneration, increasing inflammatory cells and severe epithelial necrosis in RMP administrated rats, which was evidenced by increasing level of Mercury (Hg) and Arsenic (As) from the study of ICP-MS.

Heavy metal is a critical factor in various disease and dysfunctions. Previous studies have reported that the renal proximal tubule was always the target site where heavy metal rapidly accumulated, especially Hg (Kim et al., 2010; Kumar et al., 2014). As is always closely related to cancer, cardiovascular disease and neuropathy, etc. (States et al., 2011). Results of ICP-MS demonstrated that the up-regulated As and Hg in plasma and kidney became down-regulated on 15th day after the last administration, while the concentrations of As and Hg in liver continually increased. Act as the primary organ on the detoxification of xenobiotic drugs, toxicity was accumulated in the liver.

**Figure 9:**
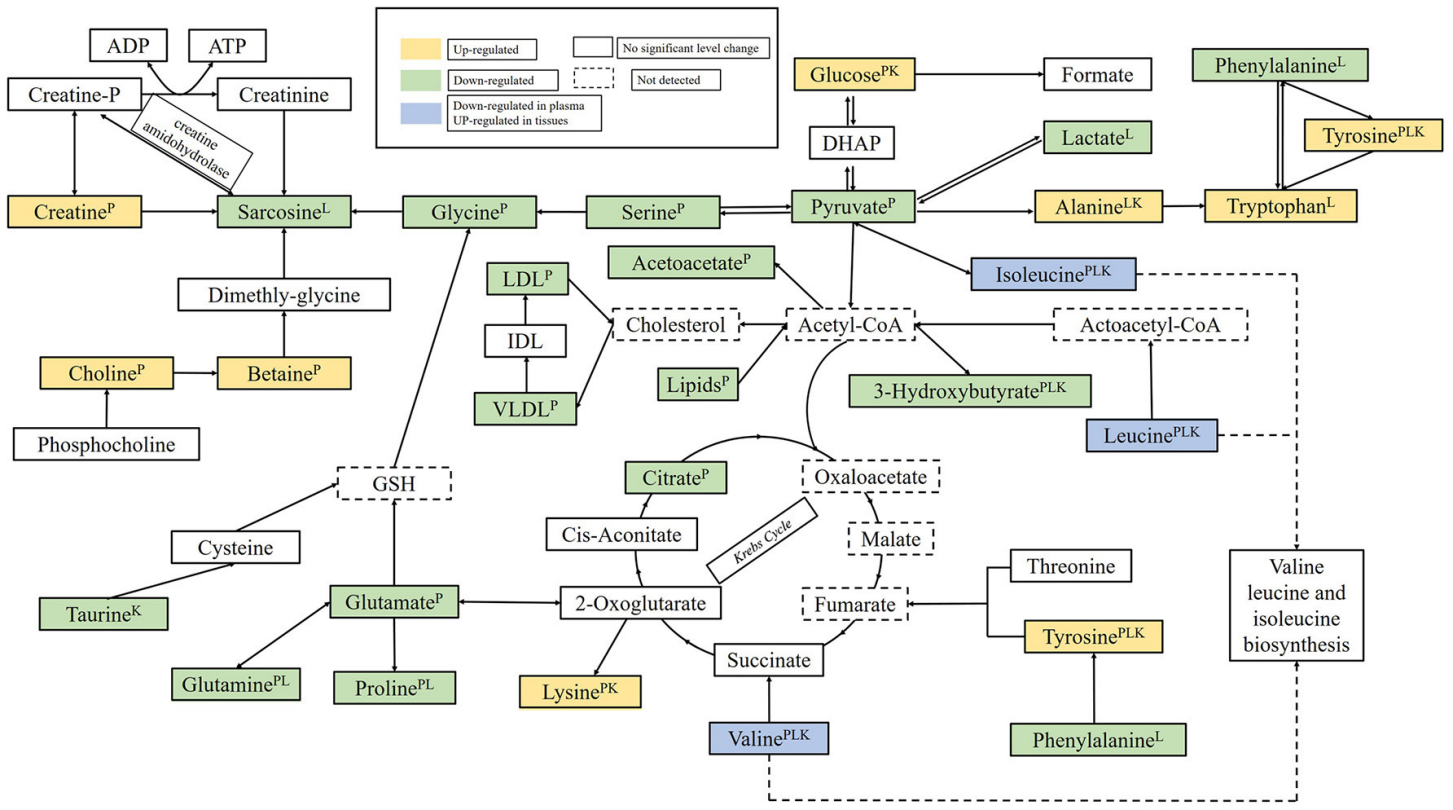
The perturbed metabolic pathways detected by NMR analysis, showing the interrelationship of the identified metabolic pathways. Metabolites with superscript “P” means that levels of metabolites from plasma were changed significantly; “L” means that levels of metabolites from liver tissue were changed significantly; “K” means that levels of metabolites from kidney tissue were changed significantly.

The biomarkers found in plasma and tissues samples were associated with important physiological functions and several metabolic pathways, such as, energy metabolism, glucose, and amino acids metabolism, etc. Based on the Human Metabolome Database (HMDB) and the KEGG pathway database (http://www.genome.jp/kegg/), schematic representation of the metabolic networks was shown in Figure 9.

TCA cycle is the start and the end of many metabolic pathways, which mainly involving the glucose aerobic oxidation, and the amino acid and fatty acids metabolisms pathways, and it harnesses the potential energy of Acetyl-CoA into the reducing power, NADH and FADH2(Krebs, 1970; Xu et al., 2013). As a crucial intermediate of glycolysis, Pyruvate can be transformed into acetyl-CoA by the pyruvate decarboxylation, and then Acetyl-CoA may be used for carrying out cellular respiration in the TCA cycle. The apparent decreasing level of lactate, pyruvate and citrate was observed on 15th day in plasma samples in RMP groups, which were in conformity with the earlier studies (Su et al., 2017), suggested that the Krebs cycle was disturbed, and the result is in consistent with the previous observation (Arakaki et al., 2008; Xu et al., 2013; Garcia-Sevillano et al., 2014). In TCA cycle, the reduction at the rate of oxidative phosphorylation and fatty acid β-oxidation may lead to a lack of acetyl-CoA in the mitochondria which will result in the abnormal levels of TCA intermediates (Xu et al., 2013). In addition, higher level of glucose and lower level of lactate in RMP group implied that the energy consumption was transferred to lipid oxidation due to the rate of reduced glycolysis (Sun et al., 2011).

Additionally, the significantly decreased levels of lipids and 3-hydroxybutate can also be observed in RMP groups. Ketone bodies, mainly 3-hydroxybutyrate, are generated from the lipolysis in the liver mitochondria (Guo et al., 2014). This indicated the reduction in energy production by the oxidation of fatty acid and that ketone body synthesis occurred to produce energy, thus used as fuel in the case of energy deficit, which was evidenced by the increased level of creatine in plasma samples. As the emergency cellular energy regulators, creatine can interact directly with ATP to produce phosphocreatine and store the energy of excess ATP (Ma et al., 2010). The higher levels of creatine suggested that RMP interfered with the creatine-phosphocreatine system an energy shortage and produced more ATP to supply energy, which generated the redundant free creatine.

Oxidative stress resulted from the imbalance between the generation of reactive oxygen species (ROS) and antioxidant defenses to counteract or detoxify their harmful effects through neutralization by antioxidants (Cuzzocrea et al., 2001; Stohs et al., 2001; Kumar et al., 2003; Wei et al., 2009). Decreased levels of plasma glutamate and glutamine were observed in the HD rats. Glutamate and glutamine are the precursor of the major natural antioxidant glutathione (GSH) and have been demonstrated to combat the oxidative injury (Guo et al., 2015). It also found a down-regulated level in kidney of RMP HD rats, which has been used to evaluate a protective action against drug-induced toxicity through antioxidant effects (Zeng et al., 2010). Therefore, the decreased levels of taurine, glutamate, and glutamine caused by RMP may induce oxidative damage.

The lower level of serum BCAAs was relative to liver damage, which can course by the elevated hormone insulin. Hormone insulin can accelerate the absorption of BCAAs in body and mainly inactivated in liver (Liao et al., 2007). When the hepatic dysfunction was caused by external influence or disease, the weakened insulin inactivation and the increased level of insulin would lead to the decreased level of BCAAs in serum (Platell et al., 2000). The reduced levels of serum BCAAs probably illustrated the liver dysfunction caused by RMP. The increase in tissues sample may suggest an inhibition of protein synthesis. And according to the previous study (Xu et al., 2013), the concentrations of BCAAs are very much related to the liver damage.

Choline is the breakdown product of phosphatidylcholine, which is the major constituent of cell membranes and lipoprotein phospholipids (Wang H. P. et al., 2013). It denoted the disruption of membrane fluidity from the increasing level of choline and the drop in serum lipids after RMP administration, which is in accordance with the previous observation of cinnabar-treated rats (Wei et al., 2008).

Betaine is a metabolite of Choline. A significant increasing level of betaine was determined in the plasma of RMP group and indicated the improved bioavailability from choline to betaine because of the inhibited choline degradation pathway (Xu et al., 2013). Previous work showed that increasing of betaine combine with the increasing choline may interfere with transmethylation pathway (Zira et al., 2013).

After 15-day recovery, the disturbed pathway reduced some of mentioned changes to a certain degree. However, for the group of high-dose RMP, it still takes time to reverse the damage.

## Conclusion

Combined the histopathological detection with ICP-MS analysis, ^1^H NMR-based metabolomics approach was used firstly to evaluate the toxicological effects of RMP. The abnormal metabolic profiles of the plasma and the tissue extracts were highlighted and could related to the perturbed metabolic pathways inducing by RMP. The identified metabolites were related to the variety of metabolic pathways including energy metabolism, amino acid metabolism and Lipid metabolism. These findings suggested that RMP may induce injury in kidney and liver and also provided several potential biomarkers for toxicity diagnostics. Metabolomics not only gains a comprehensive evaluation of the systemic response the chronic toxicity of TTM, but also holds a promise to the clinical and pharmaceutical usage.

## Author contributions

CX, CR, and JL performed the majority of the experiment; CX and JG wrote and revised the manuscript; LZ, YY, and YW supported several experiments; ZL and JC supervised the research and revised the manuscript.

### Conflict of interest statement

The authors declare that the research was conducted in the absence of any commercial or financial relationships that could be construed as a potential conflict of interest.
