# Supplementary material for: 1H NMR-Based Metabolomics Study of the Toxicological Effects in Rats Induced by “Renqing Mangjue” Pill, a Traditional Tibetan Medicine
Source: Front Pharmacol. 2017 Sep 4;8:602. doi: 10.3389/fphar.2017.00602 (PMC5591455; doi:10.3389/fphar.2017.00602)
Supplement: Supplementary file 1 [file Presentation1.PDF]

Supplementary files

^{1}H NMR-based metabolomics study of the toxicological effects in rats induced by ‘Renqing Mangjue’ pill, a Traditional Tibetan Medicine

Can Xu[1, †], Caidan Rezeng[2, †], Jian Li[3, †], Lan Zhang[1], Yujing Yan[1], Jian Gao[3], Yingfeng Wang[1], Zhongfeng Li[1*], Jianxin Chen[3*]

1 Department of Chemistry, Capital Normal University, Beijing, China

2. Research Center of Chinese and Tibetan Medicine, Medicine College of Qinghai University, Xining, China

3. Beijing University of Chinese Medicine, Beijing, China

† Can Xu, Caidan Rezeng and Jian Li contributed equally to this work.

*Correspondence should be addressed to Zhongfeng Li: lizf@cnu.edu.cn and Jianxin Chen: cjx@bucm.edu.cn

Zhongfeng Li

Department of Chemistry, Capital Normal University,

No.105 Xisanhuanbeilu, Haidian District, Beijing 100048, China

Tel: +86-10-68902655

Fax: +86-10-68902687

E-mail: lizf@cnu.edu.cn

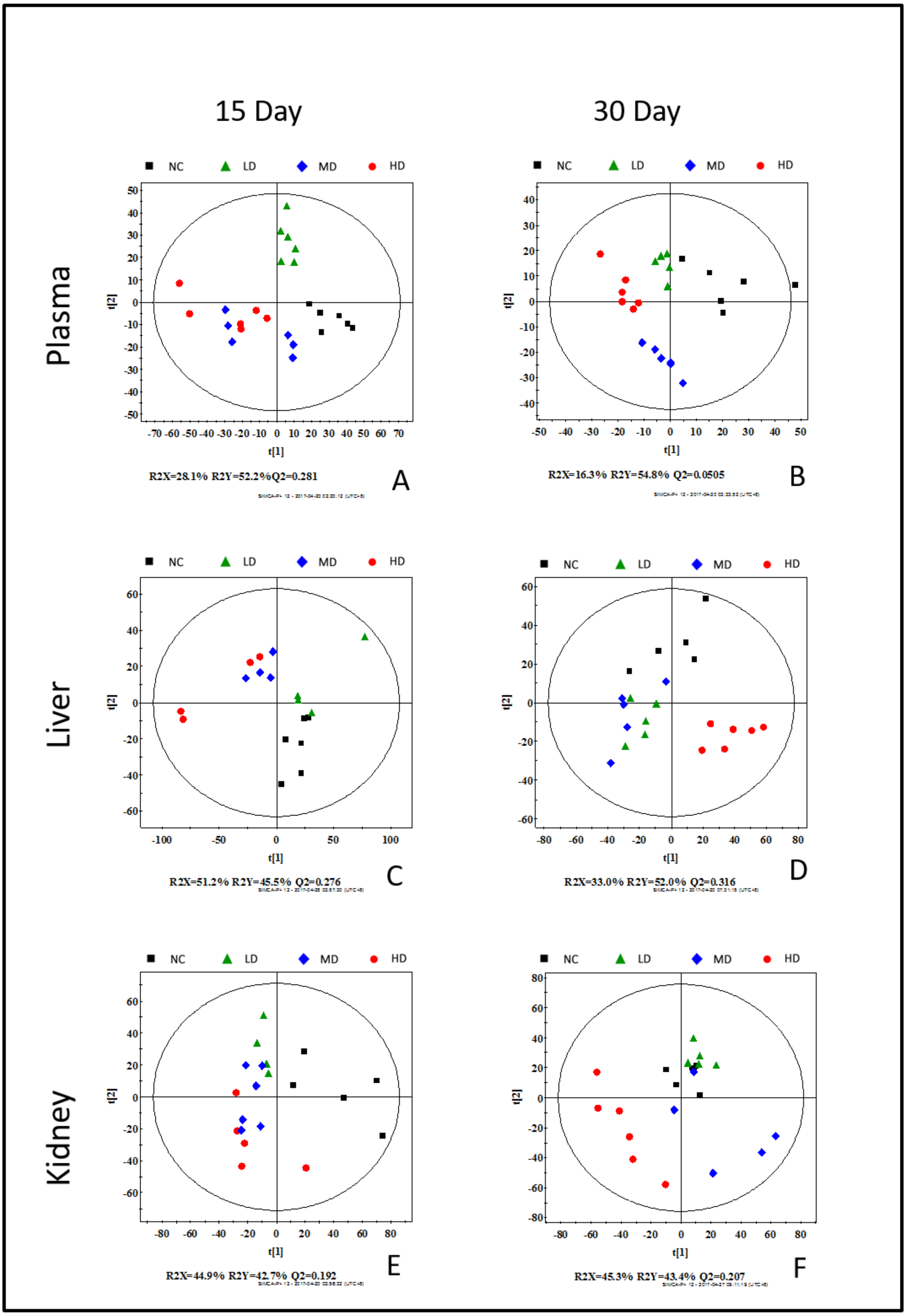

Fig. S1. Representative PLS-DA score plots (t1 vs. t2) derived from the ^{1}H NMR data of plasma (A and B), liver extract (C and D), and kidney extract (E and F) from control and dosed groups at day 15 and day 30.

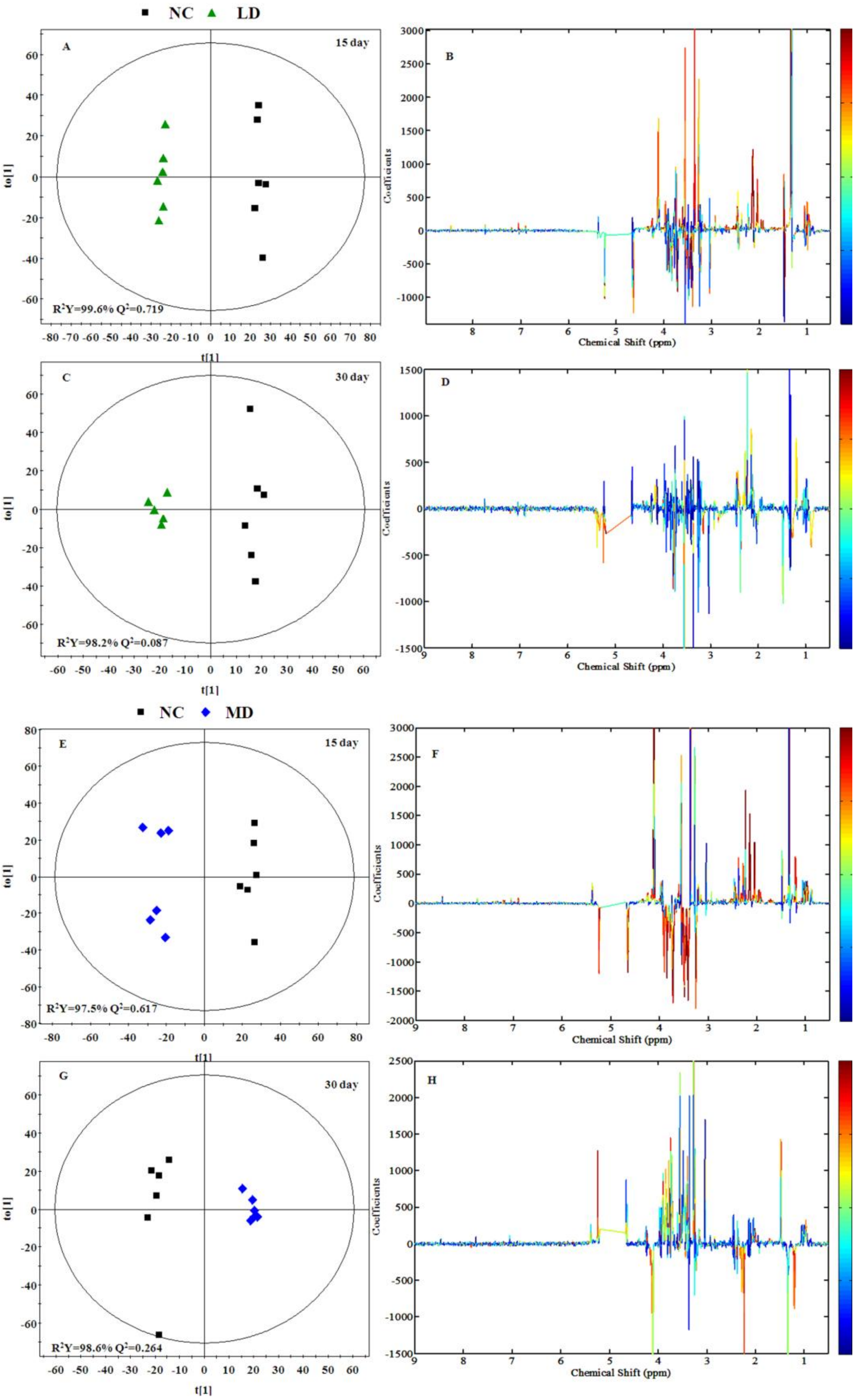

Fig. S2. OPLS-DA scores plots (A, C, E and G) and coefficient loading plots (B, D, F and H) derived from ^{1}H NMR spectra of plasma from MD/ NC and LD/ NC group at day 15 and day 30.

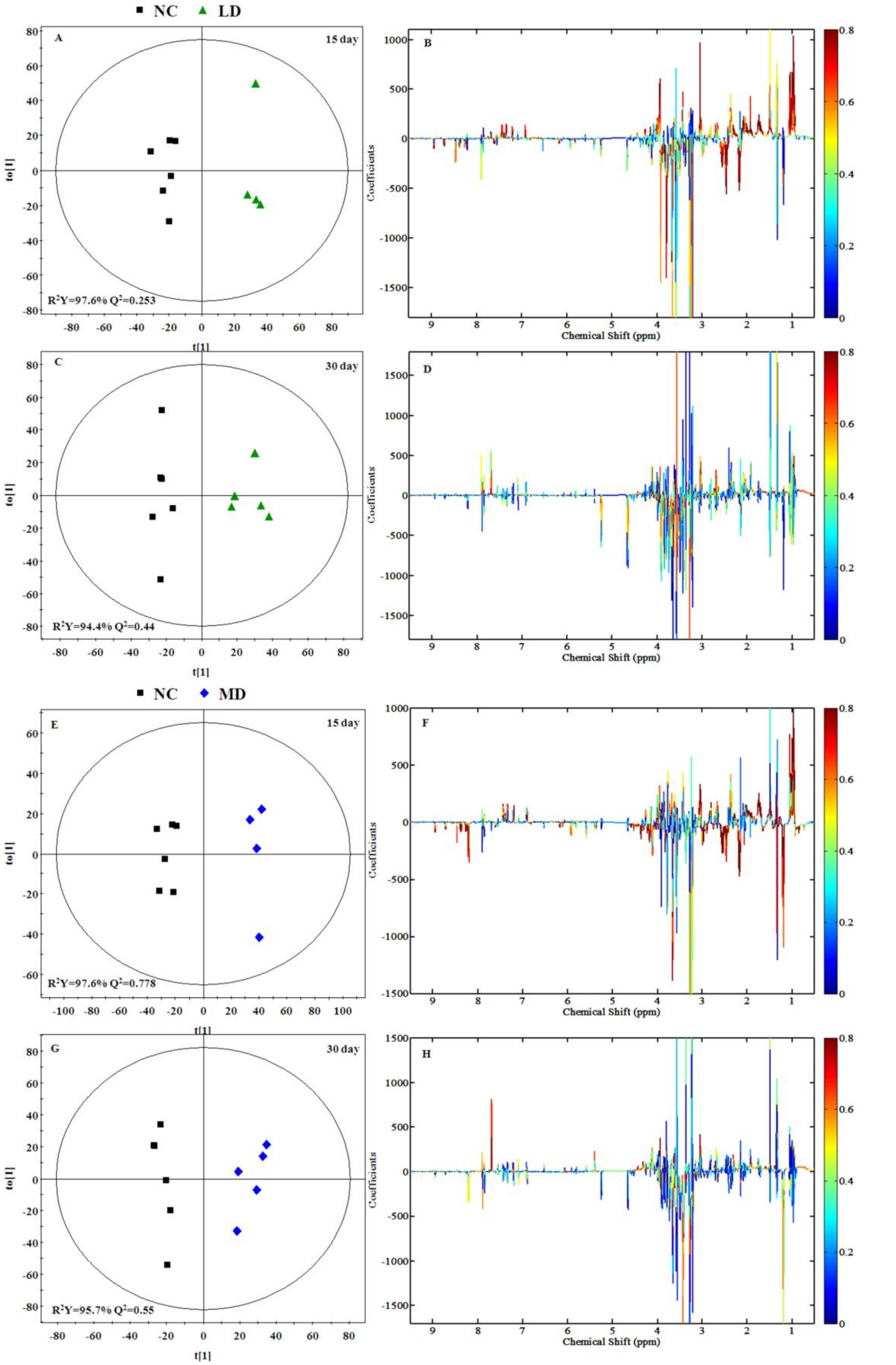

Fig. S3. OPLS-DA scores plots (A, C, E and G) and coefficient loading plots (B, D, F and H) derived from ^{1}H NMR spectra of liver from LD/ NC and MD/ NC group at day 15 and day 30.

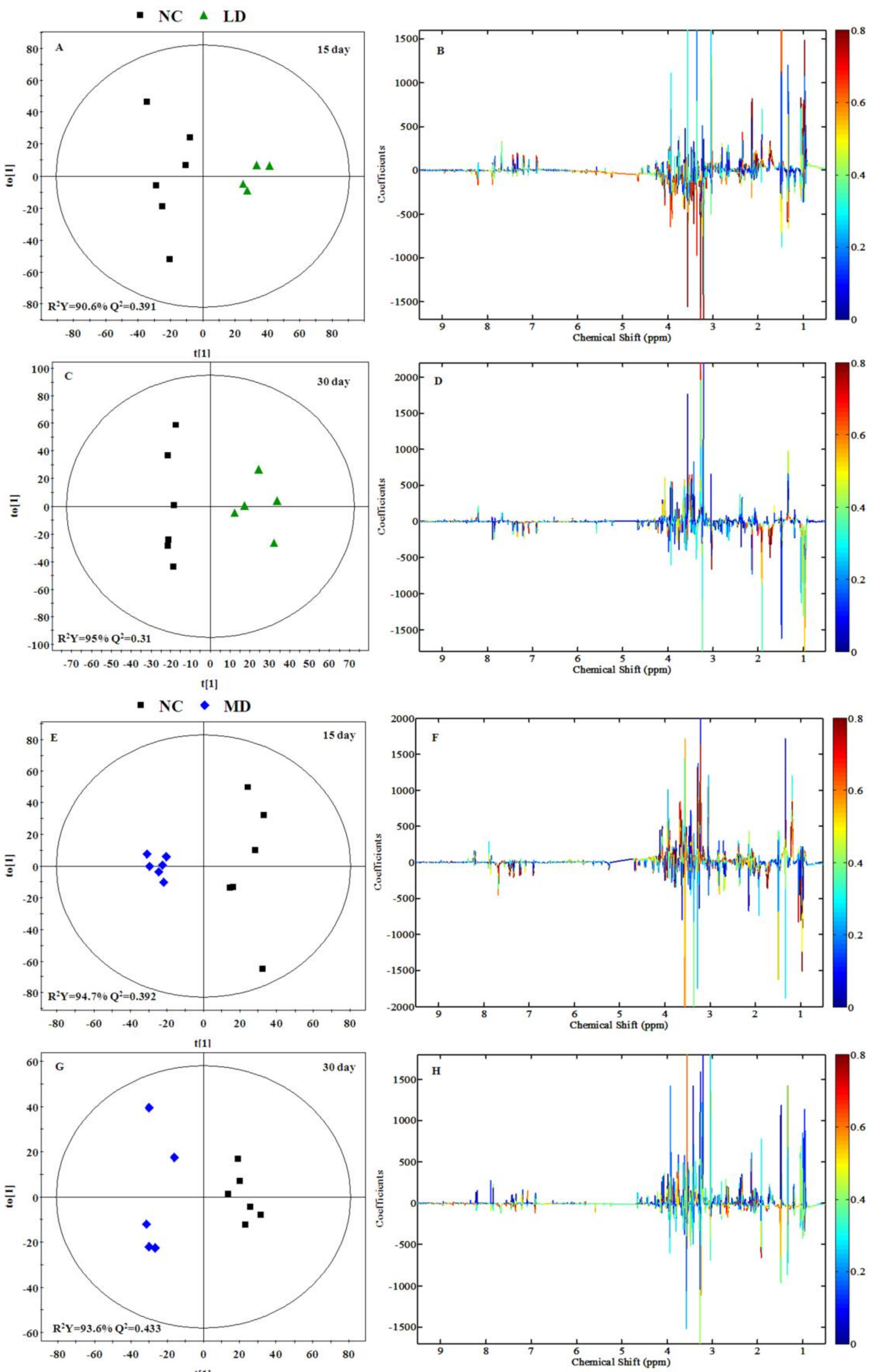

Fig. S4. OPLS-DA scores plots (A, C, E and G) and coefficient loading plots (B, D, F and H) derived from ^{1}H NMR spectra of kidney from LD/ NC and MD/ NC group at day 15 and day 30.

Table S1. Results from Pathway Analysis with MetPA from plasma.

Pathway Name	Total	Hits	Raw p	=-LOG(p)	Holm P	FDR	Impact
Valine, leucine and isoleucine biosynthesis	11	4	4.65E-06	12.279	0.000372	0.00015	0.99999
Glycine, serine and threonine metabolism	32	6	9.00E-07	13.921	7.29E-05	7.29E-05	0.53477
Methane metabolism	9	2	0.004742	5.3514	0.36035	0.054866	0.4
Glyoxylate and dicarboxylate metabolism	16	1	0.1782	1.7249	1	0.80188	0.2963
Pyruvate metabolism	22	2	0.02773	3.5852	1	0.18718	0.24337
Alanine, aspartate and glutamate metabolism	24	2	0.032665	3.4215	1	0.20353	0.14979
Aminoacyl-tRNA biosynthesis	67	7	5.54E-06	12.104	0.000437	0.00015	0.13793
Glycolysis or Gluconeogenesis	26	2	0.037921	3.2722	1	0.2194	0.12753
Citrate cycle (TCA cycle)	20	2	0.023136	3.7664	1	0.17036	0.1254
Arginine and proline metabolism	44	3	0.014424	4.2388	1	0.12982	0.07598
Cysteine and methionine metabolism	28	2	0.043484	3.1354	1	0.23481	0.04416
Starch and sucrose metabolism	23	1	0.24635	1.401	1	0.99621	0.03778
Galactose metabolism	26	1	0.2739	1.295	1	0.99621	0.03644
Primary bile acid biosynthesis	46	1	0.43473	0.83304	1	1	0.02976
Glycerophospholipid metabolism	30	1	0.30916	1.1739	1	1	0.02315
Glutathione metabolism	26	1	0.2739	1.295	1	0.99621	0.00573

Total is the total number of compounds in the pathway; the hits is the actually matched number from the user uploaded data; the raw p is the original p value calculated from the enrichment analysis; the impact is the pathway impact value calculated from pathway topology analysis.

Table S2. Results from Pathway Analysis with MetPA from liver.

Pathway Name	Total	Hits	Raw p	=-LOG(p)	Holm P	FDR	Impact
Phenylalanine, tyrosine and tryptophan biosynthesis	4	2	0.00055	7.5063	0.04342	0.01484	1
Valine, leucine and isoleucine biosynthesis	11	3	0.000125	8.9873	0.009999	0.005062	0.99999
Phenylalanine metabolism	9	2	0.003205	5.7432	0.24996	0.064893	0.40741
Tryptophan metabolism	41	1	0.3413	1.075	1	1	0.15684
Alanine, aspartate and glutamate metabolism	24	2	0.02255	3.792	1	0.30443	0.14979
Tyrosine metabolism	42	1	0.34808	1.0553	1	1	0.14045
Arginine and proline metabolism	44	2	0.068977	2.674	1	0.50792	0.064
Glycine, serine and threonine metabolism	32	1	0.27731	1.2826	1	1	0.06083
Starch and sucrose metabolism	23	1	0.20758	1.5722	1	0.98688	0.03778
Galactose metabolism	26	1	0.23149	1.4632	1	0.98688	0.03644

Total is the total number of compounds in the pathway; the hits is the actually matched number from the user uploaded data; the raw p is the original p value calculated from the enrichment analysis; the impact is the pathway impact value calculated from pathway topology analysis.

Table S3. Results from Pathway Analysis with MetPA from kidney.

Pathway Name	Total	Hits	Raw p	=-LOG(p)	Holm P	FDR	Impact
Phenylalanine, tyrosine and tryptophan biosynthesis	4	2	0.000472	7.6595	0.037252	0.012732	1
Valine, leucine and isoleucine biosynthesis	11	3	9.86E-05	9.2242	0.00789	0.003994	0.99999
Taurine and hypotaurine metabolism	8	1	0.071991	2.6312	1	0.58312	0.42857
Phenylalanine metabolism	9	2	0.002756	5.894	0.21497	0.055809	0.40741
Glycerolipid metabolism	18	1	0.15525	1.8627	1	0.96732	0.28098
Tyrosine metabolism	42	1	0.32775	1.1155	1	1	0.14045
Arginine and proline metabolism	44	1	0.34054	1.0772	1	1	0.064
Starch and sucrose metabolism	23	1	0.19424	1.6387	1	1	0.03778
Galactose metabolism	26	2	0.02276	3.7828	1	0.30726	0.03644
Primary bile acid biosynthesis	46	1	0.35311	1.041	1	1	0.02976

Total is the total number of compounds in the pathway; the hits is the actually matched number from the user uploaded data; the raw p is the original p value calculated from the enrichment analysis; the impact is the pathway impact value calculated from pathway topology analysis.

Table S4. Concentrations of As and Hg in control and dosed-group plasma and tissues sample.

sample	time points	Element	Control group	Low dosed group	Medium dosed group	High dosed group
plasma	15 day	As	6443±431.77	6679±354.41	7259±384.40*	8928±612.90**
		Hg	1.873±0.50	0.7623±0.25	1.563±0.38	1.882±0.46
	30 day	As	6832±376.31	6812±587.69	7019±642.70	8039±447.10*
		Hg	1.107±0.26	0.9108±0.08	0.8655±0.25	0.7869±0.26
liver	15 day	As	1138±93.74	1153±53.81	1142±107.50	1366±125.00
		Hg	50.37±10.82	54.78±11.28	71.39±25.48	140.5±47.47**
	30 day	As	1260±108.67	1325±156.17	1336±134.50	1424±266.25
		Hg	68.6±12.33	50.06±11.75	50.55±9.88	57.96±7.87
kidney	15 day	As	1370±177.40	1721±313.20*	1803±293.90*	2410±305.40**
		Hg	73.77±11.1	287.2±82.87**	480.6±155.70**	805.3±204.70**
	30 day	As	1466±165.22	1853±184.09*	1907±318.87**	2091±205.91**
		Hg	82.34±36.70	100.8±30.42	118.8±42.74	362.7±180.10**

Data were presented as mean±SD of n=5 animals per groups. Statistical analysis was performed by one-way ANOVA followed by Dunnett's test.
* P<0.05 versus control group. * *P<0.01 versus control group.
